# Dr. James Edwin Medden

**Published:** 1916-04

**Authors:** 


**Dr. James Edwin Medden,** Long Island College Hospital, 1895, died at Seneca Falls, of septicaemia Feb. 11, aged 56. He had been a supervisor for Seneca Co. and president of the County Medical Society.

